# Cross‑Cultural Validation of the Chronic Rhinosinusitis Patient-Reported Outcomes (CRS-PRO) Questionnaire in Portuguese

**DOI:** 10.7759/cureus.74875

**Published:** 2024-11-30

**Authors:** André Sousa-Machado, Mariana Cascao, Patricia Sousa, Taciano Rocha, Antonio Castanheira, Leigh Sowerby

**Affiliations:** 1 Otolaryngology, Local Health Unit of Trás-os-Montes and Alto Douro (ULSTMAD), Vila Real, PRT; 2 Medical Education and Simulation, Faculty of Health Sciences, University of Beira Interior, Covilhã, PRT; 3 Clinical Research, Victoria Hospital, Ontario, CAN; 4 Rhinology and Anterior Skull Base Surgery, University of Western Ontario, Ontario, CAN

**Keywords:** chronic rhinosinusitis, cross-cultural validation, patient-reported outcomes, portuguese, questionnaire

## Abstract

Introduction

Chronic rhinosinusitis (CRS) presents with different clinical patterns with variable responses to treatment. Clear criteria for specifying disease severity and assessing symptom control are lacking in the current literature. We aimed to perform a cross-cultural adaptation of the chronic rhinosinusitis patient-reported outcomes (CRS-PRO), creating a Portuguese version to use as a routine questionnaire in the evaluation of patients with CRS.

Methods

The CRS-PRO questionnaire was translated according to the recommendations of the International Society for Pharmacoeconomics and Outcomes Research (ISPOR) through a three-step procedure including a backward translation.

Results

After translation completion, the questionnaire was evaluated in 40 participants (23 men) who completed the questionnaire on two separate occasions in 1.4 minutes (SD 0.615). Twenty of them were patients with CRS (60% with polyps), and the other 20 were healthy subjects who were considered a control group. The average age of the study participants was 43 years old (SD 16). The intraclass correlation coefficient (ICC) values for the CRS with nasal polyps (CRSwNP) group ranged from 0.65 to 0.89, indicating good to excellent reliability across the 12 items. All ICC values were statistically significant (p < 0.01).

Conclusion

This study presents the Portuguese version of the CRS-PRO questionnaire, an adapted, validated, and well-accepted instrument for evaluating CRS symptoms in the Portuguese-speaking population.

## Introduction

Chronic rhinosinusitis (CRS) with nasal polyps (CRSwNP) manifests in various clinical forms and severity [1]. There is a noted lack of definitive criteria for assessing disease severity and control within the existing literature [2]. Treatment possibilities for CRSwNP are broadening, and attention has been increasingly focused on the subjective impact of symptoms, assessed via patient-reported outcome measures (PROMs). Although the Sinonasal Outcome Test-22 (SNOT-22) is popular in clinical trials, some specialists believe the questions are too broad and not specific to CRS, leading to exaggerated disease severity by comorbidities. Also, with 22 items, it is a time-consuming tool that can be challenging in a busy practice and can lead to survey fatigue in patients [3]. Currently, no assessment tool includes functional, physical, or radiological magnitude domains and effects on quality of life (QoL) or symptom changes during treatment of CRSwNP [4].

The newly introduced 12-item CRS-patient-reported outcomes (CRS-PRO) is a validated questionnaire responsive to medical and surgical treatments for patients diagnosed with both CRSwNP and non-polyp CRS (CRSsP). This measure is simple to administer and has proven validity for assessing CRS symptoms, as noted by Lin et al. Compared to the longer tools such as the SNOT-22, CRS-PRO more accurately reflects radiographic changes post-medical management or endoscopic sinus surgery (ESS) and is divided into three subdomains: physical symptoms, sensory impairment, and psychosocial effects [5,6].

The translation and validation of questionnaires into other languages is a critical step to verify their validity and applicability, ensuring conceptual consistency with the original version. This study discusses the process of cultural adaptation and validation of the original CRS-PRO questionnaire into Portuguese for consistent use in evaluating patients with CRS.

## Materials and methods

The CRS-PRO is owned by Bruce K. Tan and is protected under copyright laws as intellectual property. Permission for using the CRS-PRO questionnaire was obtained from the development team. All participants gave their oral informed consent before their involvement in the study, which adhered to the ethical standards of the Declaration of Helsinki.

To ensure privacy, all collected data were anonymized, and no identifiable information was stored. The questionnaire, originally developed in US English, needed to be translated into Portuguese, following the guidelines of the International Society for Pharmacoeconomics and Outcomes Research (ISPOR) [7].

The translation process involved three main steps: forward translation, backward translation, and patient testing.

Forward translation

Initially, three bilingual Portuguese-speaking staff from both Brazil and Portugal independently translated the questionnaire from English to Portuguese. They then collaborated to merge their translations into one version that was conceptually equivalent to the original, ensuring the language was colloquial and comprehensible. This collaborative effort produced the first Portuguese version.

Backward translation

This first Portuguese version was translated back into English by two native English speakers fluent in Portuguese who had not been exposed to the original questionnaire. This step aimed to identify any discrepancies or errors. The backward translations were evaluated and refined following feedback from Dr. Tan, resulting in a second Portuguese version. This version was then assessed by a panel of health professionals and patients for clarity and ease of use, leading to the creation of the third Portuguese version.

Patient testing

The third version was then validated with 30 patients across various regions in Portugal to ensure that the instructions, methodology, and questionnaire items were clear and unambiguous. Based on the participants' feedback during these face-to-face interviews, further modifications were made, culminating in the fourth Portuguese version. After additional proofreading and final adjustments, the fifth version was finalized and tested for validity and reliability as presented in this current study.

The study ensured that all questionnaires were completed, with no concerns about missing data. The reproducibility of this new scale was evaluated through test-retest reliability assessed in all patients who completed the questionnaire again under the same conditions after 15 days.

The consistency of the responses was measured using intraclass correlation coefficients (ICCs), with values closer to 1 indicating higher repeatability. Statistical analyses were conducted using R software, with p-values determined through two-sided tests.

The time interval between the two administrations of 15 days was designed to be short enough to prevent significant changes in the constructs being measured, yet long enough to avoid recall effects. The test-retest reliability of the 12-item CRS-PRO questionnaire was assessed using ICCs, a two-way mixed effects model, with an absolute agreement (IBM SPSS Statistics v29.0, IBM Corp., Armonk, NY).

## Results

The dataset comprised responses from 40 participants (23 men) who completed the questionnaire on two separate occasions, in 1.4 minutes (SD 0.615). Twenty of them were patients with CRS (60% with polyps), and the other 20 were healthy subjects who were considered the control group. The average age of the study participants was 43 years (SD 16). Four patients with CRS presented with asthma, and all were in the CRSwNP subgroup. Lund-Mackay score was also assessed in the group with CRS, with a mean value of 13.05 (SD 7.08).

Table 1 presents the ICC values comparing responses from the first and second administrations of the questionnaire among CRS patients with nasal polyps (CRSwNP) and healthy control participants.

**Table 1:** Reliability analysis of CRS-PRO questionnaire. Test-retest analysis of 12 questionnaire items among the CRSwNP patients and healthy participants. * p = 0.01. Additional significances were <0.0001. N/A: Not applicable. CRS-PRO: Chronic rhinosinusitis patient-reported outcomes.

	All participants (n = 40)				CRS (n = 20)				Control (n = 20)			
Test x Retest	ICC	95% CI			ICC	95% CI			ICC	95% CI		
Item 1	0.97	0.94	to	0.99	0.87	0.39	to	0.96	0.95	0.87	to	0.98
Item 2	0.98	0.97	to	0.99	0.97	0.92	to	0.99	0.93	0.82	to	0.97
Item 3	0.97	0.94	to	0.99	0.90	0.71	to	0.96	1.00	N/A		
Item 4	0.97	0.95	to	0.99	^*^0.67	0.17	to	0.87	1.00	N/A		
Item 5	0.99	0.99	to	1.00	0.99	0.97	to	1.00	1.00	N/A		
Item 6	0.95	0.89	to	0.97	0.81	0.43	to	0.93	1.00	N/A		
Item 7	0.99	0.98	to	1.00	0.96	0.89	to	0.98	1.00	N/A		
Item 8	0.99	0.97	to	0.99	0.82	0.55	to	0.93	1.00	N/A		
Items 9 to 12	1.00	N/A			1.00	N/A			1.00	N/A		

The ICC values for the CRSwNP group ranged from 0.65 to 0.89, indicating good to excellent reliability across the 12 items. The ICC values for the control group were higher, ranging from 0.78 to 0.95, also reflecting good to excellent reliability. All ICC values were statistically significant at the p < 0.01 level or lower.

These results suggest that the questionnaire shows good reliability over time for both CRS patients and healthy controls based on the ICC analyses. The control group showed particularly high reliability, with ICCs consistently in the excellent range above 0.75. Overall, the questionnaire appeared reliable for assessing chronic sinusitis outcomes, with responses remaining generally stable between the two time points for both study groups.

The authors also explored the content validity of the CRS-PRO questionnaire with some questions listed in Table 2. These questions helped the authors check the suitability of the questionnaire for the population in question.

**Table 2:** Face and content validity assessment of the CRS-PRO questionnaire in Portuguese CRS-PRO: Chronic rhinosinusitis patient-reported outcomes.

	CRS (n = 20)		Control (n = 20)	
	No	Yes	No	Yes
Any emotional difficulties in the questionnaire interpretation?	12 (60)	8 (40)	20 (100)	N/A
Do you have any worsening symptoms such as congestion, runny nose, reduced sense of smell, headaches, or cough?	15 (75)	5 (25)	20 (100)	N/A
Have you ever answered a similar questionnaire?	14 (70)	6 (30)	20 (100)	N/A
Any interpretation difficulties related to the language of this questionnaire?	19 (95)	1 (5)	19 (95)	1 (5)
Did you find the questions confusing?	18 (90)	2 (10)	15 (75)	5 (25)
Time for questionnaire completion (min), Mean (SD)	1.4 (0.615)			

The Portuguese version of the CRS-PRO questionnaire is shown in Table 3.

**Table 3:** CRS-PRO questionnaire in Portuguese version. CRS-PRO: Chronic rhinosinusitis patient-reported outcomes.

CRS-PRO - Por favor, responda cada uma das seguintes perguntas sobre como sua rinossinusite crónica o(a) afeta. Nos últimos 7 dias…					
Physical symptoms	Nada	Um pouco	Alguma coisa	Bastante	Muito
1) Tive dificuldade em respirar pelo nariz	0	1	2	3	4
2) Senti pressão na face	0	1	2	3	4
3) Tive dores na face	0	1	2	3	4
4) Tive que assoar o nariz	0	1	2	3	4
5) Tenho tido tosse	0	1	2	3	4
6) Tive muco na garganta	0	1	2	3	4
7) Tive muco no nariz	0	1	2	3	4
Problemas Sensoriais	Nada	Um pouco	Alguma coisa	Bastante	Muito
8) Tive problemas com o meu olfato	0	1	2	3	4
Efeitos Psicossociais	Nada	Um pouco	Alguma coisa	Bastante	Muito
9) Os meus sintomas mantiveram-me acordado(a) durante a noite	0	1	2	3	4
10) Senti-me fatigado	0	1	2	3	4
11) Tive receio de que a minha condição venha a piorar	0	1	2	3	4
12) Senti-me frustrado(a) com minha condição	0	1	2	3	4

## Discussion

In this research, a cross-cultural adaptation of a new questionnaire (CRS-PRO) was made to measure specific to chronic rhinosinusitis [5]. This tool effectively gauges the symptoms and psychosocial effects of CRS, including its two primary clinical variants, CRSwNP, and CRS without nasal polyps [7-9].

Similar methodologies have been employed by other research teams [10-13]. It has been noted that significant cultural differences can exist within populations previously thought to be homogeneous [14]. Therefore, the reversed translation technique employed was crucial to capture an accurate reflection of the original content [14]. This technique allowed us to translate the underlying concepts or ideas, rather than performing a direct word-for-word translation of each item. Representation from both Brazil and Portugal was employed to ensure that jargon and idioms unique to each respective country were not included in the translation and that universal understanding was available.

The ISPOR guidelines for cross-cultural adaptation enabled us to develop a culturally adapted Portuguese version of the CRS-PRO equivalent to the original English version [8]. According to these guidelines, at least two translators are recommended for the English-to-Portuguese translation, followed by a meeting to establish consensus. We engaged three independent translators to ensure that the concept behind the questionnaire was translated as accurately as possible, enhancing reproducibility.

According to cross-cultural adaptation guidelines, assembling a panel of experts to evaluate the questionnaire is advisable [15]. In step 2, Portuguese version 2 was reviewed by a panel of three healthcare professionals and 10 patients. This phase was vital for refining the questionnaire and underscoring the importance of including diverse perspectives, such as that of physicians and patients, to counter potential biases from medical professionals about the clarity of technical terms [16,17].

The test-retest reliability metric confirmed the consistency and reproducibility of the Portuguese version outcomes, validating the effectiveness and fidelity of the translated questionnaire. This demonstrates that the translation-back translation method did not compromise its validity. While no translation is flawless and some conceptual differences may persist, when retranslated into English, these were deemed minimal and did not alter the overall meaning or comprehension of the questionnaire [17,18].

The main limitations of this study are the sample size, possible conceptual differences in translations, and focus on participants from a specific geographical region.

## Conclusions

The Portuguese version of the CRS-PRO questionnaire, now validated and adapted, is a valuable tool for assessing CRS symptoms and QoL among Portuguese-speaking populations. This tool proves particularly useful in the follow-up of CRS patients, enabling the evaluation of outcomes from medical and surgical treatments in CRS patients. Also, it facilitates the comparison of results with international studies and within clinical trials.
